# Ten-year results of the PORTEC-2 trial for high-intermediate risk endometrial carcinoma: improving patient selection for adjuvant therapy

**DOI:** 10.1038/s41416-018-0310-8

**Published:** 2018-10-25

**Authors:** B. G. Wortman, C. L. Creutzberg, H. Putter, I. M. Jürgenliemk-Schulz, J. J. Jobsen, L. C. H. W. Lutgens, E. M. van der Steen-Banasik, J. W. M. Mens, A. Slot, M. C. Stenfert Kroese, B. van Triest, H. W. Nijman, E. Stelloo, T. Bosse, S. M. de Boer, W. L. J. van Putten, V. T. H. B. M Smit, R. A. Nout, for the PORTEC Study Group

**Affiliations:** 1 Department of Radiation Oncology, Leiden University Medical Center, Leiden, The Netherlands; 2 Department of Medical Statistics, Leiden University Medical Center, Leiden, The Netherlands; 3 Department of Radiation Oncology, University Medical Center Utrecht, Utrecht, The Netherlands; 4 Department of Radiotherapy, Medisch Spectrum Twente, Enschede, The Netherlands; 5 Maastricht Radiation Oncology Clinic, Maastricht, The Netherlands; 6 Radiotherapy Group, Arnhem, The Netherlands; 7 Department of Radiation Oncology, Erasmus MC- Cancer Institute, Rotterdam, The Netherlands; 8 Radiotherapy Institute Friesland, Leeuwarden, The Netherlands; 9 Radiotherapy Group, Deventer, The Netherlands; 10 Department of Radiotherapy, Netherlands Cancer Institute, Amsterdam, The Netherlands; 11 Department of Gynaecologic Oncology, University Medical Center Groningen, Groningen, The Netherlands; 12 Department of Pathology, Leiden University Medical Center, Leiden, The Netherlands; 13 Department of Biostatistics, ErasmusMC Cancer Institute, Rotterdam, The Netherlands

## Abstract

**Background:**

PORTEC-2 was a randomised trial for women with high-intermediate risk (HIR) endometrial cancer, comparing pelvic external beam radiotherapy (EBRT) with vaginal brachytherapy (VBT). We evaluated long-term outcomes combined with the results of pathology review and molecular analysis.

**Methods:**

427 women with HIR endometrial cancer were randomised between 2002–2006 to VBT or EBRT. Primary endpoint was vaginal recurrence (VR). Pathology review was done in 97.4%, combined with molecular analysis.

**Results:**

Median follow-up was 116 months; 10-year VR was 3.4% versus 2.4% for VBT vs. EBRT (*p* = 0.55). Ten-year pelvic recurrence (PR) was more frequent in the VBT group (6.3% vs. 0.9%, *p* = 0.004), mostly combined with distant metastases (DM). Ten-year isolated PR was 2.5% vs. 0.5%, *p* = 0.10, and DM 10.4 vs. 8.9% (*p* = 0.45). Overall survival for VBT vs. EBRT was 69.5% vs. 67.6% at 10 years (*p* = 0.72). L1CAM and p53-mutant expression and substantial lymph-vascular space invasion were risk factors for PR and DM. EBRT reduced PR in cases with these risk factors.

**Conclusion:**

Long-term results of the PORTEC-2 trial confirm VBT as standard adjuvant treatment for HIR endometrial cancer. Molecular risk assessment has the potential to guide adjuvant therapy. EBRT provided better pelvic control in patients with unfavourable risk factors.

## Introduction

Women with endometrial cancer (EC) are often diagnosed at early stage of disease, and in general have a favourable prognosis^1^. Randomised trials have shown that adjuvant radiation therapy (RT) for stage I EC significantly reduced the risk of locoregional recurrence, without difference in overall survival^2–5^. High-intermediate risk (HIR) factors were defined in both the PORTEC-1 and GOG#99 trials to identify women who were at relatively higher risk of recurrence^2,4^. As the majority of recurrences in these trials were located in the vaginal vault, the Post-Operative Radiation Therapy in Endometrial Cancer trial (PORTEC)-2 trial was initiated in 2002 to investigate the efficacy of vaginal brachytherapy (VBT) as compared to pelvic RT (EBRT) for women with stage I EC with HIR factors to maximise local control, with reduced toxicity and better quality of life. Five-year results of the PORTEC-2 trial showed equally low rates of vaginal recurrence in both treatment arms, without differences in overall and disease-free survival^6^. Higher rates of treatment-related toxicity, especially gastro-intestinal symptoms with impact on health-related quality of life (HRQL), were recorded in the EBRT arm, while patients who received VBT reported HRQL and symptoms scores which did not differ from those of an age-matched norm population^7–9^. As a result, VBT became standard adjuvant treatment for women with HIR endometrial carcinoma.

More recently, novel molecular risk factors in endometrial cancer were described. In 2013 the Cancer Genome Atlas (TCGA) group published results of an extensive genomic characterisation of endometrial cancer, defining four different molecular subgroups with distinct prognosis: a *POLE*-ultramutated group; a microsatellite-unstable hypermutated group; a copy-number-low group and a copy-number-high group driven by *TP53* mutation^10^. *POLE*-ultramutated EC had very favourable outcomes, while those with *TP53* mutation had an unfavourable prognosis. For the copy-number-low group, no specific driver mutation was identified. Analysis of these four molecular subgroups by their surrogate markers (p53 expression by immunohistochemistry; PCR based determination of microsatellite instability (MSI); and analysis of POLE exonuclease domain mutations by Sanger sequencing) in more than 900 paraffin-embedded (FFPE) tissue samples of the PORTEC-1 and PORTEC-2 biobank, led to a useful and practical molecular classification tool for the clinic. Results of these analyses confirmed the prognostic significance of these 4 molecular subgroups, which was confirmed in a similar analysis reported by Talhouk et al.^11,12^. Moreover, several other strong clinicopathologic and molecular risk factors such as substantial (diffuse or multifocal) lymph-vascular space invasion (LVSI), L1CAM expression and beta catenin mutation were analysed. A molecular integrated risk profile was defined which was able to distinguish favourable, intermediate and unfavourable subgroups within the group of HIR EC, with a clear difference in outcomes^11–14^.

With current knowledge of molecular risk features, the question remains whether patient selection for vaginal brachytherapy can be further improved, thereby decreasing both over- and undertreatment. It was hypothesised that a small subgroup of patients with unfavourable risk features such as *TP53* mutation, L1CAM expression ( > 10%), or substantial LVSI might have had better pelvic control if they had received EBRT. The present analysis was done to analyse long-term outcomes of the PORTEC-2 trial, and evaluate whether specific clinicopathologic and molecular risk factors can be used to determine optimal adjuvant treatment for subgroups at higher risk of recurrence.

## Material and methods

### Patient selection and eligibility criteria

The PORTEC-2 trial was a multicentre randomised trial, which recruited patients between May 2002 and September 2006. Women were eligible if they had been diagnosed with endometrial carcinoma with high-intermediate risk factors (HIR) and were randomly allocated to either vaginal brachytherapy (VBT) or pelvic radiotherapy (EBRT). HIR was defined as either (1) FIGO 1988 stage 1C (≥50% myometrial invasion) with age greater than 60 and grade 1 or 2; or (2) FIGO 1988 stage 1B (<50% myometrial invasion) with age greater than 60 and grade 3; or (3) FIGO 1988 stage 2A (endocervical glandular involvement, which is stage I in FIGO 2009) with any age, except for grade 3 with deep invasion. Exclusion criteria were: serous or clear cell carcinoma; staging lymphadenectomy; > 8 weeks interval between surgery and radiotherapy; history of previous malignancy; previous radiotherapy, hormonal or chemotherapy; Crohn’s disease or ulcerative colitis. Detailed information on patient selection, randomisation and masking, treatment and follow-up was described previously^6^. The primary endpoint of the study was vaginal recurrence (VR). Secondary endpoints were pelvic recurrence (PR), distant recurrence (DR), overall survival, endometrial cancer-related survival (CSS), disease-free survival (DFS), and toxicity and quality of life. The trial protocol was approved by the Dutch Cancer Society (CKTO 2001–04) and the Ethics Committees of participating centres. Written informed consent was given by all patients.

### Treatment and follow-up

Radiation therapy was administered within 8 weeks after total abdominal hysterectomy and bilateral salpingo-oophorectomy. Lymphadenectomy was not performed routinely^15,16^. In case of suspicious lymph nodes found at surgery, these were selectively removed. EBRT was delivered to the pelvic area using a total dose of 46 Gy in 2 Gy daily fractions, five times per week. The clinical target volume consisted of the proximal vagina, parametria, and internal, external and caudal common iliac lymph node regions up to the level of the promontory. Treatment planning was performed by CT-based three-dimensional conformal planning using multiple fields with individual shielding; usually a 4-field box technique.

VBT was delivered with a vaginal cylinder to the proximal half of the vagina, with dose specification at 5 mm distance from the surface of the cylinder. High-dose rate (HDR) equipment was used in 85%, delivering a dose of 21 Gy in 3 fractions of 7 Gy, with an interval of 1 week; 15% received an equivalent dose using LDR (0.5-0.7 Gy/hr) or MDR (1 Gy/hr) equipment^6^.

Follow-up consisted of alternating visits to the patient’s gynaecologist and radiation oncologist every 3–4 months in the first 3 years, at 6 month intervals in the 4^th^ and 5^th^ years, and yearly thereafter, up to at least 7 years. If needed, follow-up information was obtained from the GP and the national population registry at 10 years after treatment. At follow-up visits, physical examination was performed and side-effects or recurrence of disease were reported and treated. Patient-reported health-related quality of life and symptoms were recorded by the EORTC QLQ-C30 and specific symptom modules for bladder, bowel and sexual symptoms. Short-term and long-term quality of life outcomes have been reported separately^7–9^.

### Pathology review and analysis of molecular characteristics

Central pathology review was performed by specialised gynaeco-pathologists, after the patient had been included in the trial. More recently, comprehensive analysis of molecular alterations has been done, in a translational research project using the pooled PORTEC-1 and PORTEC-2 biobank^11^. TCGA molecular subgroups were assessed using surrogate markers on FFPE tissue samples^11,12^. Immunohistochemical techniques and DNA analysis were used to assess polymerase-epsilon (*POLE*) mutations; microsatellite instability (MSI); and p53 protein expression (scored as p53-wildtype/mutant/null staining)^11^. In addition, analysis of L1CAM expression, with > 10% expression being L1CAM positive, and the presence and quantification of LVSI were assessed, according to the methods described previously^13,14^. Based on previous analyses, only substantial LVSI was taken into account, since mild (focal) LVSI was not associated with increased risk of recurrence^14^.

### Statistical analysis

All randomised patients were kept in the analyses for primary and secondary endpoints, which was by intention to treat. Analysis of molecular risk factors was performed only in those patients whose high-intermediate risk features were confirmed at central pathology review (confirmed-HIR). For these analyses of confirmed-HIR cases, data of the previous study on the molecular risk factors within the PORTEC-1 and 2 biobank were used (selecting the PORTEC-2 cases only)^11^.

Time-to event analyses were done with log-rank tests and Cox proportional hazards regression models with date of randomisation as starting point. Both log-rank tests and Cox regression models were stratified for FIGO stage but were essentially the same with and without adjustment, and results are presented without adjustment. Overall survival (OS) was calculated from date of randomisation to death from any cause, with censoring at date of last information for patients alive. Endometrial cancer-related survival (CSS) was calculated from date of randomisation to date of death related to endometrial cancer, with censoring of patients who died of other causes and of patients alive at date of last follow-up or last information, respectively. Disease-free survival (DFS) was calculated from date of randomisation to date of disease recurrence or to date of death from any cause, with censoring of patients alive and recurrence-free. Data for patients who were alive and recurrence-free were censored at date of last follow-up or of information on vital status. The competing risk method (with death as competing risk) was used for analysis of vaginal, and pelvic recurrence and distant metastasis. First failure type was vaginal recurrence when an isolated vaginal recurrence had occurred; pelvic recurrence in case of pelvic recurrence with or without vaginal recurrence; and first failure type was distant if a distant recurrence was diagnosed, with or without pelvic or vaginal recurrence. The Kaplan-Meier method was used for OS, CSS and DFS. Analyses of (molecular) risk factors were done using univariable Cox proportional hazard models^11^. Risk factors with a *p*-value below 0.1 in univariable analysis were included in multivariable analysis. Chi-square and Fisher’s exact tests were used to compare (molecular) risk factors between treatment groups. SPSS was used to perform statistical analyses, version 23.0 (IBM SPSS, Inc., Chicago, IL).

## Results

**Fig. 1:**
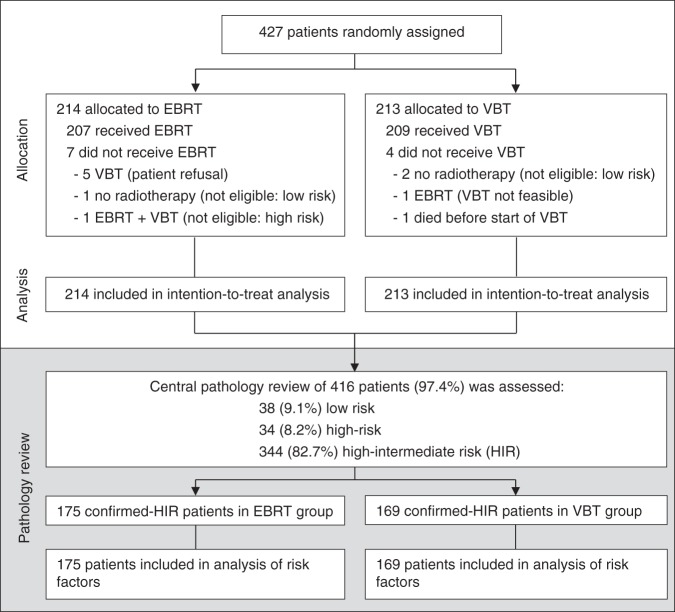
Consort diagram of the PORTEC-2 trial with pathology review

In total, 427 women with HIR endometrial carcinoma were included in the trial; 214 were randomly assigned to receive EBRT and 213 to VBT (Fig. 1). Patient and tumour characteristics were equally distributed over the two treatment groups (Table 1). The database was frozen on May 1^st^ 2016 and by then, the median follow-up was 116 months (range 18–163 months).

**Table 1:** Patient and tumour characteristics and pathology review

Intention to treat population				
*n* = 427	EBRT (*n* = 214)		VBT (*n* = 213)	
**Age**				
</=60 years	8	3.7%	8	3.8%
60-70 years	109	50.9%	99	46.5%
>70 years	97	45.3%	106	49.8%
**FIGO 1988 stage**				
IB	19	8.9%	16	7.5%
IC	172	80.4%	171	80.3%
IIA	23	10.7%	26	12.2%
**Myometrial invasion**				
< 50%	31	14.5%	28	13.1%
> 50%	183	85.5%	185	86.9%
**Grade**				
1	99	46.3%	103	48.4%
2	97	45.3%	94	44.1%
3	18	8.4%	16	7.5%
**LVSI**				
Present	25	11.7%	21	9.9%
Absent	189	88.3%	192	90.1%

Confirmed-HIR at pathology review				
*n* = 344	EBRT (*n* = 175)		VBT (*n* = 169)	
**Grade**				
1	148	84.6%	134	79.3%
2	16	9.1%	23	13.6%
3	11	6.3%	12	7.1%
**Substantial LVSI**				
Yes	9	5.1%	7	4.1%
No	160	91.4%	156	92.3%
Missing^a^	6	3.4%	6	3.6%
**Molecular subgroup**				
POLE	10	5.7%	6	3.6%
MSI	41	23.4%	36	21.3%
NSMP	103	58.9%	96	56.8%
TP53^b^	10	5.7%	15	8.9%
Double classifiers	4	2.3%	6	3.6%
Missing^a^	7	4.0%	10	5.9%
**L1CAM expressio** **n** ^c^				
> 10%	4	2.3%	14	8.3%
< 10%	168	96.0%	151	89.3%
Missing^a^	3	1.7%	4	2.4%

^a^Material not appropriate for test or failed test

^b^As assessed by p53 protein expression

^c^Significant difference (*p* = 0.010)

**Fig. 2:**
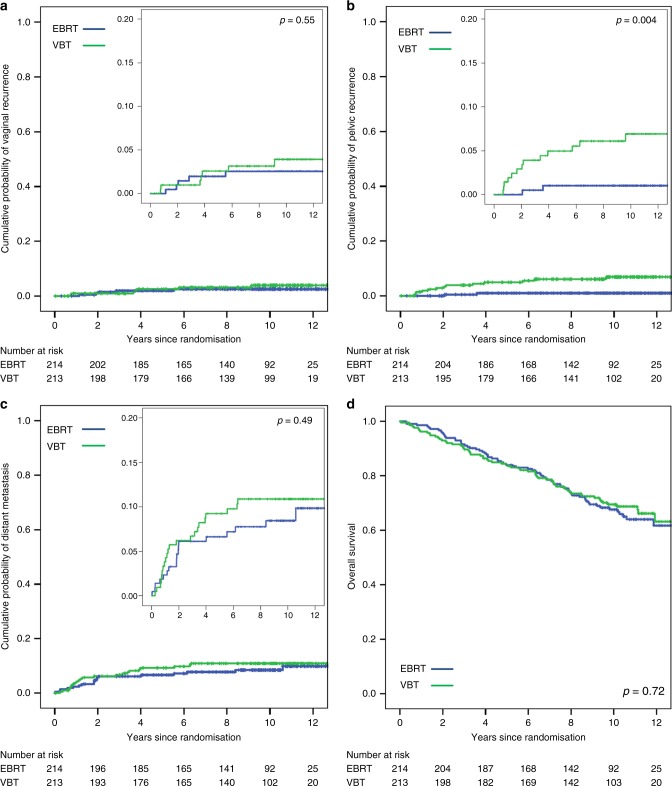
Ten year results of the PORTEC-2 trial

Long-term results of the intention to treat analysis are presented in Table 2 and Fig. 2. A total of 12 women developed a vaginal recurrence, seven in the VBT group and five in the EBRT group. The 10-year vaginal recurrence rates were 3.4% and 2.4% for VBT and EBRT, respectively (*p* = 0.55). Pelvic recurrences were diagnosed in 13 women in the VBT group and two in the EBRT group, with 10-year rates of 6.3% vs. 0.9% (*p* = 0.004); of these, 2.5% vs. 0.5% were isolated pelvic recurrences (*p* = 0.10). Ten-year rates of distant metastases were 10.4% vs. 8.9% for VBT vs EBRT (*p* = 0.45). No significant differences in first failure types were found, except for simultaneous distant and pelvic recurrence: 3.6% in the VBT group versus 0.5% in the EBRT group, *p* = 0.03. Long-term results of the confirmed-HIR population are shown in the Supplementary Data Table 2. In this population the 10-year vaginal recurrence rates were 2.7% vs. 3.1% (*p* = 0.78) and the pelvic recurrence rates 7.4% and 1.2% (*p* = 0.01) for VBT vs. EBRT, respectively.

**Table 2:** Long-term outcomes (intention to treat population)

	EBRT (*n* = 214)			VBT (*n* = 213)			HR (95% CI)	
	Events	5-year %	10-year %	Events	5-year %	10-year %	VBT:EBRT	*p* value
**First failure type**								
Vaginal recurrence	3	1.1%	1.5%	5	0.9%	3.0%	1.68 (0.40 - 7.03)	0.47
Pelvic recurrence	1	0.5%	0.5%	5	1.4%	2.5%	5.07 (0.59 - 43.41)	0.10
Distant recurrence	18	6.6%	8.9%	22	8.9%	10.4%	1.25 (0.67 - 2.33)	0.49
Distant alone	15	5.7%	7.0%	13	5.5%	6.6%	0.88 (0.42 - 1.86)	0.75
Distant and pelvic	1	0.5%	0.5%	7	3.0%	3.6%	7.16 (0.88 - 58.23)	0.03
Distant and vaginal	2	0.5%	1.1%	1	0.5%	0.5%	0.51 (0.05 - 5.65)	0.58
**Total failure**								
Vaginal recurrence	5	1.9%	2.4%	7	2.4%	3.4%	1.42 (0.45 - 4.46)	0.55
Pelvic recurrence	2	0.9%	0.9%	13	4.6%	6.3%	6.65 (1.50 - 29.48)	0.004
Distant recurrence	18	6.6%	8.9%	22	8.9%	10.4%	1.25 (0.67 - 2.33)	0.49
**Endometrial cancer-related survival**	18	93.2%	90.9%	23	91.7%	88.2%	1.29 (0.70 - 2.39)	0.42
**Disease-free survival**	71	82.1%	68.0%	72	81.2%	66.7%	1.03 (0.74 - 1.43)	0.87
**Overall survival**	70	84.0%	67.6%	66	84.0%	69.5%	0.94 (0.67 - 1.32)	0.72

A total of 136 women died during follow-up: 70 in the VBT group and 66 in the EBRT group. Cause of death was endometrial carcinoma in 30.1%; secondary cancer in 13.2%; and intercurrent disease in 50.7%. Ten-year overall survival was 69.5% vs. 67.6% (*p* = 0.72) and 10-year endometrial cancer-related survival 88.2% vs. 90.9% (*p* = 0.42) for VBT vs. EBRT groups, respectively.

### Prognostic factors

**Fig. 3:**
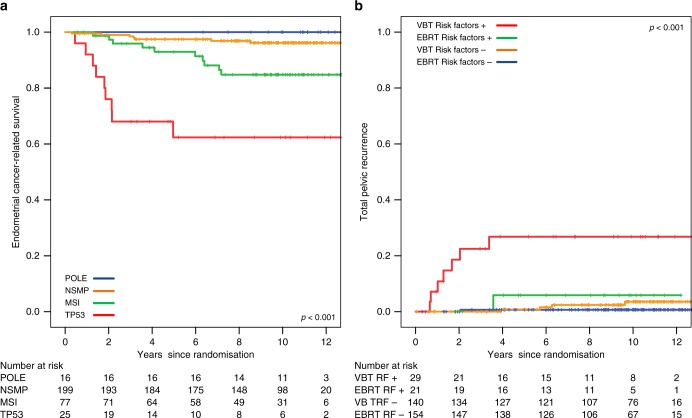
**a** Endometrial cancer-related survival by 4 molecular subgroups. **b** Total pelvic recurrence by unfavourable risk factors (LVSI, p53-mutant or L1CAM expression)

Central pathology review was available for 416 patients (97.4%). HIR status was confirmed by the review gynaeco-pathologist in 344 cases (82.7%), while 34 were determined high risk (8.2%) and 38 low risk (9.1%), see Table 1 and Fig. 1. Figure 3a shows the CSS for the four molecular subgroups in confirmed HIR patients. For women with tumours harbouring a *POLE* mutation, 10-year CSS was 100%, in contrast to 96.2% for no specific molecular profile, 84.8% for MSI and 62.3% for p53-mutant tumours (*p* < 0.001).

Among confirmed HIR patients a subgroup of 50 women presented with any of the unfavourable risk features substantial LVSI, p53-mutant and/or L1CAM expression; 17.2% in the VBT group and 12% in the EBRT group, *p* = 0.18. L1CAM expression was found in 14 VBT patients, vs. four in the EBRT arm (*p* = 0.010). Rates of substantial LVSI and p53-mutant expression did not differ significantly between the treatment arms, see Table 1. Eleven patients had both p53–mutant and L1CAM expression, and three had both LVSI and L1CAM expression.

Multivariable analysis of unfavourable risk factors in confirmed HIR patients is presented in Table 3 and similar results were found in all patients with material available (Supplementary Data Table 2). Substantial LVSI was found to be a very strong independent risk factor for pelvic and distant recurrence (hazard ratios 8.73 (*p* = 0.005) and 5.36 (*p* = 0.001), respectively) and for endometrial cancer-related survival (HR 7.16, *p* < 0.001). L1CAM expression (HR 4.18, *p* = 0.016) and p53-mutant expression (HR 3.35, *p* = 0.015) were significant prognostic factors for distant recurrence and CSS (HR 5.05, *p* = 0.006 and HR 3.30, *p* = 0.015). Main findings of multivariable analysis for DFS were similar to those of CSS (data not shown). Although significant in univariable analysis, L1CAM and p53-mutant expression did not reach significance for pelvic recurrence in multivariable analysis. Figure 3b and Supplementary Data Fig. 1 show that the higher risk of total pelvic recurrence in the VBT group is restricted to patients with unfavourable features.

**Table 3:** Multivariable analysis of recurrence in confirmed-HIR patients

		Pelvic recurrence (total)		Distant recurrence		Endometrial cancer-related survival	
	No. ^a^	HR (95% CI)	*p* value	HR (95% CI)	*p* value	HR (95% CI)	*p* value
**Treatment group**							
EBRT	163	1	0.054	1	0.805	1	0.740
VBT	154	4.58 (0.97 - 21.52)		0.91 (0.41 - 2.00)		0.87 (0.40 - 1.94)	
**LVSI**							
no/mild	301	1	0.005	1	0.001	1	< 0.001
substantial	16	8.73 (1.95 - 39.22)		5.36 (1.91 - 15.07)		7.16 (2.71 - 18.91)	
**TP53** ^b^							
wild type	288	1	0.065	1	0.015	1	0.015
mutation	29	3.82 (0.92 - 15.83)		3.35 (1.27 - 8.84)		3.30 (1.26 - 8.64)	
**L1CAM**							
< 10%	300	1	0.126	1	0.016	1	0.006
> 10%	17	3.79 (0.69 - 20.93)		4.18 (1.31 - 13.33)		5.05 (1.59 - 16.06)	

^a^Total no. 317; 27 cases had insufficient material for analysis of all factors

^b^ As assessed by p53 protein expression

## Discussion

The present analysis of long-term results of the PORTEC-2 trial confirmed the excellent vaginal control with adjuvant vaginal brachytherapy for women with high-intermediate risk endometrial cancer, with 10-year vaginal control above 96% in both arms. Although the risk of pelvic recurrence was significantly (6% vs. 1%) higher in the VBT group, the majority of these women presented with simultaneous distant metastasis, resulting in similarly low rates of isolated pelvic recurrence in both treatment arms. Moreover, no differences were found in 10-year rates of distant metastasis and overall survival. As previously reported, low toxicity rates and better health-related quality of life were found among women who received VBT compared to EBRT, even after more than 7 years^9^. Similar findings were reported in a Swedish trial comparing EBRT combined with VBT versus VBT alone for women with intermediate-risk endometrial cancer^17^. The 5-year locoregional relapse rates were 1.5% vs. 5% (*p* = 0.013), with crude rates of vaginal recurrence of 1.9% vs. 2.7%, and quality of life results favoured VBT. These long-term findings confirm VBT as the adjuvant treatment of choice for women with early stage endometrial cancer with high-intermediate risk features.

Implementation of HIR risk factors as determined in both the PORTEC-1 and GOG#99 trials for the indication for adjuvant radiotherapy reduced the number of women who received radiotherapy by 50% at the time, sparing them unnecessary and potentially toxic treatment^2–4,18^. In the PORTEC-1 trial, the 5-year risk of vaginal recurrence among women with high-intermediate risk features was reduced from 15% without radiotherapy, to 2% with EBRT. Although the risk of vaginal recurrence was subsequently found in the PORTEC-2 trial to be similarly low with VBT, it can be argued that this still represents overtreatment, as 8 women need to be treated to prevent one vaginal recurrence, and selection for adjuvant treatment could be improved^19^. Moreover, EBRT might have provided better pelvic control for the few (6%) patients who developed pelvic recurrence after VBT, even if the majority presented with simultaneous distant metastases. These results indicate there is a clear need for additional risk factors that improve the current risk classification.

Both the TCGA analysis and studies determining the molecular subgroups by their surrogate markers indicated that distinguishing the four molecular subgroups had strong prognostic significance^10–12^. Mutation of the tumour suppressor gene *TP53* has been related to early tumour progression in multiple cancer types as well as in endometrial cancer, and is associated with grade 3 and with non-endometrial (mostly serous) histology, while *POLE* mutation leads to only rare recurrence and excellent outcomes^20^. MSI is an intermediate risk factor but associated with Lynch syndrome and might have therapeutic implications. More recently MSI detection has been replaced by analysis of mismatch repair deficiency (MMRd), and detection of MLH-1 promotor hypermethylation in those with MMRd^21^.

Substantial LVSI and L1CAM expression are strong risk factors for recurrence^10,11,13,14^. L1CAM is a cell adhesion molecule and mediates cell motility, is associated with epithelial mesenchymal transition and early disease spread. Several large series have confirmed the negative prognostic impact of L1CAM expression^13,22,23^. Interestingly, while there is some overlap between *TP53* mutation and L1CAM expression, L1CAM has been shown to be an independent risk factor, frequently associated with, but independent from *TP53* mutation^24^. This was confirmed in the current analysis, where 38% of L1CAM positive patients did not have p53-mutant expression, and 63% of patients with p53-mutant expression did not have L1CAM expression. LVSI has long been known for its adverse prognostic impact, being associated with the risk of (microscopic) nodal metastases and with higher rates of recurrence and lower CSS, both in the presence and absence of lymph node metastases^25,26^. A recent large study using the pooled PORTEC biobank in which LVSI was quantified and graded as absent, mild (a single focus or few foci) or substantial (diffuse or multifocal) showed that substantial LVSI is a highly significant risk factor for pelvic and distant recurrence^14^.

In this long-term analysis, substantial LVSI, p53-mutant and L1CAM expression were all strongly associated with the risk of pelvic recurrence, distant metastasis and endometrial cancer-related survival. Moreover, in the small subgroup of women with high-intermediate risk endometrial cancer with any of these unfavourable risk factors, EBRT provided a significantly better pelvic control than VBT.

Strengths of this study are the uniform and random allocation of treatment, long and complete follow-up and central pathology review in 97% of patients. Central pathology review was performed because various studies had shown frequent inter-observer variation within the field of gynaecopathology, with a poor reproducibility especially of the intermediate grade^27–29^. More recent analysis of the inter-observer variability in the pathology review of the PORTEC-3 trial, which was required before randomisation, showed that in 43% of all patients at least one of the pathology items changed, with grade (20%) and histological type (15%) being the most frequent items of disagreement. Upfront pathology review resulted in 8% of all patients being ineligible for the trial^30^.

Analysis of the long-term results within the population that was confirmed-HIR after pathology review in the PORTEC-2 trial ( > 80%) showed no significant differences compared to the intention to treat analysis, possibly also because a similar number of patients were deemed either low (38) or high risk (34) at pathology review (Fig. 1, Supplementary Data Table 2).

These long-term results show that among the large group of women with early stage endometrial cancer with risk features, the subgroup of patients with unfavourable risk factors is small, and that the combination of clinicopathologic and molecular factors adequately select the women who might benefit from EBRT or more intensive treatment. This is supported by the fact that more pelvic recurrences occurred in the VBT group, in which more patients with p53-mutant expression and with L1CAM expression were found compared to the EBRT group (Table 1).

The potential benefit of adjuvant chemotherapy to decrease disease recurrence in women with early stage, high-intermediate or high-risk endometrial cancer has been subject of several trials, which did not show differences in overall and relapse-free survival compared to EBRT^31,32^. In the GOG249 trial, 601 women with stage I-II endometrial cancer with risk factors (deep invasion, grade 3 or serous/clear cell histology) were randomised to pelvic EBRT vs. VBT with three cycles of carboplatin/paclitaxel chemotherapy. Recently presented 5-year results showed no differences in relapse- free and overall survival^33^. However, even though 89% had lymphadenectomy and were node negative, pelvic and para-aortic failures were significantly more frequent after VBT and chemotherapy, while acute toxicity was increased, leading to the conclusion that EBRT remains the standard adjuvant treatment for early stage, high-risk disease. This finding again suggests that results of adjuvant EBRT are similar with and without lymphadenectomy, as was also seen in the GOG#99 and PORTEC-1 trial^2,4^, and that detecting microscopic nodal involvement, similar to extensive LVSI, seems a marker but not a cause of distant spread. Previous randomised trials have not shown any survival benefit from lymphadenectomy in early stage disease^15,16^. The strength of the molecular markers is that they may more individually predict if specific tumours might be at risk of early disease spread. Therefore, an integrated clinicopathologic and molecular risk profile has the potential to guide adjuvant treatment and could distinguish the few women with HIR endometrial cancer who would benefit from EBRT instead of standard VBT^11,12^.

In the currently ongoing PORTEC-4a trial, women with stage I-II EC with high-intermediate risk features are randomised to receive adjuvant treatment directed by their integrated molecular risk profile or standard vaginal brachytherapy^34^. The molecular profile stratifies patients into favourable (about 50%) who will be observed, intermediate risk (about 45%) who will receive brachytherapy, and an unfavourable group (about 5%) who will receive EBRT, and thus aims to further refine risk stratification, reduce over- and undertreatment and increase cost-effectiveness. The PORTEC-4a trial was shown to be feasible by evaluation of the pilot phase, with a satisfactory patient acceptance rate and feasibility of performing the molecular assessment within 2 weeks^35^.

In conclusion, long-term results of PORTEC-2 confirmed VBT as the adjuvant treatment of choice for women with high-intermediate risk endometrial cancer. EBRT might provide better pelvic control in the small subgroup of women with unfavourable risk factors (substantial LVSI, L1CAM expression or p53*-*mutant expression).

## Electronic supplementary material

Supplementary Data Figure 1

Supplementary Data Table 1

Supplementary Data Table 2
