# Supplementary material for: Ten-year results of the PORTEC-2 trial for high-intermediate risk endometrial carcinoma: improving patient selection for adjuvant therapy
Source: Br J Cancer. 2018 Oct 25;119(9):1067–74. doi: 10.1038/s41416-018-0310-8 (PMC6219495; doi:10.1038/s41416-018-0310-8)
Supplement: Supplementary file 2 — Supplementary Data Table 1 [file 41416_2018_310_MOESM2_ESM.docx]

| Supplementary Data Table 1. Multivariable analysis of recurrence in patients with material available (no 416) | | | | | | | | | | |
| --- | --- | --- | --- | --- | --- | --- | --- | --- | --- | --- |
|  |  | **Pelvic recurrence (total)** | | | **Distant recurrence** | | | **Endometrial cancer-related survival** | | |
|  | **No. ^1^** | HR (95% CI) | | p-value | HR (95% CI) | | p-value | HR (95% CI) | | p-value |
| **Treatment group** | |  |  |  |  |  |  |  |  |  |
| EBRT | 198 | 1 | | 0.025 | 1 | | 0.627 | 1 | | 0.859 |
| VBT | 189 | 5.76 (1.25 - 26.54) | |  | 1.18 (0.61 - 2.28) | |  | 1.07 (0.54 -2.12) | |  |
| **LVSI** |  |  |  |  |  |  |  |  |  |  |
| no/mild | 367 | 1 | | 0.005 | 1 | | < 0.001 | 1 | | < 0.001 |
| substantial | 20 | 10.08 (2.42 - 41.89) | |  | 6.31 (2.65 - 15.03) | |  | 6.94 (2.89 - 16.76) | |  |
| **TP53^2^** |  |  |  |  |  |  |  |  |  |  |
| wild type | 347 | 1 | | 0.065 | 1 | | 0.039 | 1 | | 0.023 |
| mutation | 40 | 2.71 (0.73 - 10.12) | |  | 2.38 (1.05 - 5.41) | |  | 2.66 (1.15 - 6.17) | |  |
| **L1CAM** |  |  |  |  |  |  |  |  |  |  |
| < 10% | 355 | 1 | | 0.126 | 1 | | 0.002 | 1 | | 0.001 |
| > 10% | 32 | 4.43 (1.01 - 19.34) | |  | 4.09 (1.71 - 9.75) | |  | 4.74 (1.94 - 11.61) | |  |
| **^1^** Total no 387; 29 cases had insufficient material for analysis of all factors | | | | | | |  |  |  |  |

**^2^** As assessed by p53 protein expression
