# Supplementary material for: Ten-year results of the PORTEC-2 trial for high-intermediate risk endometrial carcinoma: improving patient selection for adjuvant therapy
Source: Br J Cancer. 2018 Oct 25;119(9):1067–74. doi: 10.1038/s41416-018-0310-8 (PMC6219495; doi:10.1038/s41416-018-0310-8)
Supplement: Supplementary file 3 — Supplementary Data Table 2 [file 41416_2018_310_MOESM3_ESM.docx]

| Supplementary Data Table 2. Long-term outcomes for the confirmed-HIR patients | | | | | |  |  |  |  |
| --- | --- | --- | --- | --- | --- | --- | --- | --- | --- |
|  |  | **EBRT (N=175)** | | | **VBT (N=169)** | | | **HR (95% CI)** | |
|  |  | Events | 5-year % | 10-year % | Events | 5-year % | 10-year % | VBT:EBRT | p-value |
| **First failure type** | |  |  |  |  |  |  |  |  |
| Vaginal recurrence | | 3 | 1.8% | 1.8% | 3 | 1.3% | 2.1% | 1.04 (0.21 - 5.13) | 0.97 |
| Pelvic recurrence | | 1 | 0.6% | 0.6% | 4 | 1.3% | 3.0% | 4.10 (0.46 - 36.74) | 0.17 |
| Distant recurrence | | 14 | 6.4% | 8.6% | 16 | 8.0% | 10.0% | 1.20 (0.58 - 2.45) | 0.63 |
| distant alone | | 11 | 5.2% | 6.8% | 8 | 3.7% | 5.2% | 0.76 (0.31 - 1.89) | 0.55 |
| distant and pelvic | | 1 | 0.6% | 0.6% | 7 | 3.8% | 4.5% | 7.38 (0.91 - 59.96) | 0.03 |
| distant and vaginal | | 2 | 0.6% | 1.3% | 1 | 0.6% | 0.6% | 0.53 (0.05 - 5.79) | 0.59 |
| **Total failures** | |  |  |  |  |  |  |  |  |
| Vaginal recurrence | | 5 | 2.4% | 3.1% | 4 | 1.9% | 2.7% | 0.83 (0.22 - 3.09) | 0.78 |
| Pelvic recurrence | | 2 | 1.2% | 1.2% | 11 | 5.0% | 7.4% | 5.77 (1.28 - 26.03) | 0.01 |
| Distant recurrence | | 14 | 6.4% | 8.6% | 16 | 8.0% | 10.0% | 1.20 (0.58 - 2.45) | 0.63 |
| **Endometrial cancer-related survival** | | 14 | 93.5% | 91.4% | 16 | 93.3% | 89.7% | 1.18 (0.57 - 2.41) | 0.66 |
| **Disease-free survival** | | 55 | 82.7% | 68.3% | 57 | 82.2% | 67.6% | 1.05 (0.72 - 1.52) | 0.80 |
| **Overall survival** | | 55 | 85.0% | 67.8% | 52 | 85.2% | 70.4% | 0.94 (0.64 - 1.37) | 0.74 |
